# Associations of insulin resistance and insulin secretion with bone mineral density and osteoporosis in a general population

**DOI:** 10.3389/fendo.2022.971960

**Published:** 2022-09-20

**Authors:** Yi-Hsiu Fu, Wei-Ju Liu, Chia-Lin Lee, Jun-Sing Wang

**Affiliations:** ^1^ Department of Education, Taichung Veterans General Hospital, Taichung, Taiwan; ^2^ Department of Medical Research, Taichung Veterans General Hospital, Taichung, Taiwan; ^3^ Division of Endocrinology and Metabolism, Department of Internal Medicine, Taichung Veterans General Hospital, Taichung, Taiwan; ^4^ School of Medicine, National Yang Ming Chiao Tung University, Taipei, Taiwan; ^5^ Department of Post-Baccalaureate Medicine, College of Medicine, National Chung Hsing University, Taichung, Taiwan; ^6^ Rong Hsing Research Center for Translational Medicine, Institute of Biomedical Science, National Chung Hsing University, Taichung, Taiwan

**Keywords:** bone mineral density, insulin, insulinemia, insulin resistance, osteoporosis

## Abstract

We investigated the associations of insulin resistance and β-cell secretion with bone mineral density (BMD) and osteoporosis using data from the National Health and Nutrition Examination Survey. Data on BMD assessed using dual-energy x-ray absorptiometry from 5292 participants were analyzed. Insulin resistance and β-cell secretion were assessed using the Homeostatic Model Assessment for Insulin Resistance (HOMA-IR) and β-cell function (HOMA-β), respectively. We divided the study population into four groups according to HOMA-IR (<2 vs. ≥ 2) and HOMA-β (<100 vs. ≥ 100). BMD and T score at the lumbar spine, hip joint, and femur were used for analyses. Osteoporosis was defined as a T score ≤ -2.5. Logistic regression analyses were conducted to examine the associations of HOMA-IR and HOMA-β with osteoporosis, and the joint effects of HOMA-IR and HOMA-β on osteoporosis. We found a positive association between HOMA-IR and osteoporosis in participants with a HOMA-β ≥ 100 (OR 8.773, 95% CI 2.160-35.637, p=0.002 at the femoral neck). A negative association between HOMA-β and osteoporosis was noted in those with a HOMA-IR <2 (OR 0.183, 95% CI 0.038-0.882, p=0.034 at the femoral neck). Compared with participants who had HOMA-IR <2 and HOMA-β <100, those with HOMA-IR <2 and HOMA-β ≥ 100 had a lower risk of osteoporosis (OR 0.126, 95% CI 0.020-0.805, p=0.032 at the femoral neck). In conclusion, the association between HOMA-β and BMD/osteoporosis changed as HOMA-IR increased. HOMA-β was negatively associated with osteoporosis when HOMA-IR <2. The association was not significant when HOMA-IR ≥ 2.

## Introduction

Osteoporosis is characterized by low bone mass and abnormal microstructure, leading to bone fragility and susceptibility to fracture (1). The diagnosis of osteoporosis is based on an assessment of bone mineral density (BMD), which can be conducted using a modality such as dual-energy x-ray absorptiometry (DXA) (2, 3). Osteoporosis is defined as a BMD T score of ≤ -2.5 (2, 3). The likelihood of osteoporosis tends to increase as a function of age, and therefore the incidence of osteoporotic fractures has increased markedly worldwide (4, 5). It has been predicted that the number of people at high risk of osteoporotic fracture in 2040 will be doubled the number of people at high risk in 2010 (5). This may lead to a substantial health care burden and even an excess risk of mortality (5–7).

Although BMD has been used to diagnose osteoporosis, its clinical use might be limited as most fragility fractures occur in people with a BMD T score > -2.5 (8, 9). For example, patients with type 2 diabetes are at an increased risk of fracture (10, 11); however, they have a higher BMD than those without the disease (12, 13). This may be partly explained by the association of bone fragility with the pathogenesis of diabetes (14). Moreover, insulinemia and insulin resistance (IR) might have conflicting effects on bone mass. Insulinemia has been associated with a higher BMD (15, 16), while IR has been inversely associated with bone mass (17, 18). Findings in previous studies are not consistent (15–20).

The complex effects of insulinemia and IR on bone mass are not yet clear. In this study, we aimed to investigate the associations of IR and pancreatic β-cell secretion with BMD and osteoporosis using cross-sectional data from the National Health and Nutrition Examination Survey (NHANES).

## Materials and methods

This study was conducted using publicly available data from the NHANES (https://www.cdc.gov/nchs/nhanes/index.htm), which is a multistage, cross-sectional, nationwide evaluation of health parameters in the U.S. conducted by the National Center for Health Statistics. Data on BMD assessed using DXA were available, and thus we investigated the associations of IR and pancreatic β-cell secretion with BMD and osteoporosis in this population. Our study protocol was approved by the Institutional Review Board of Taichung Veterans General Hospital, Taichung, Taiwan (approval number: CE18312A). We conducted this study in accordance with the Declaration of Helsinki, and all the NHANES participants provided informed consent.

**Figure 1:**
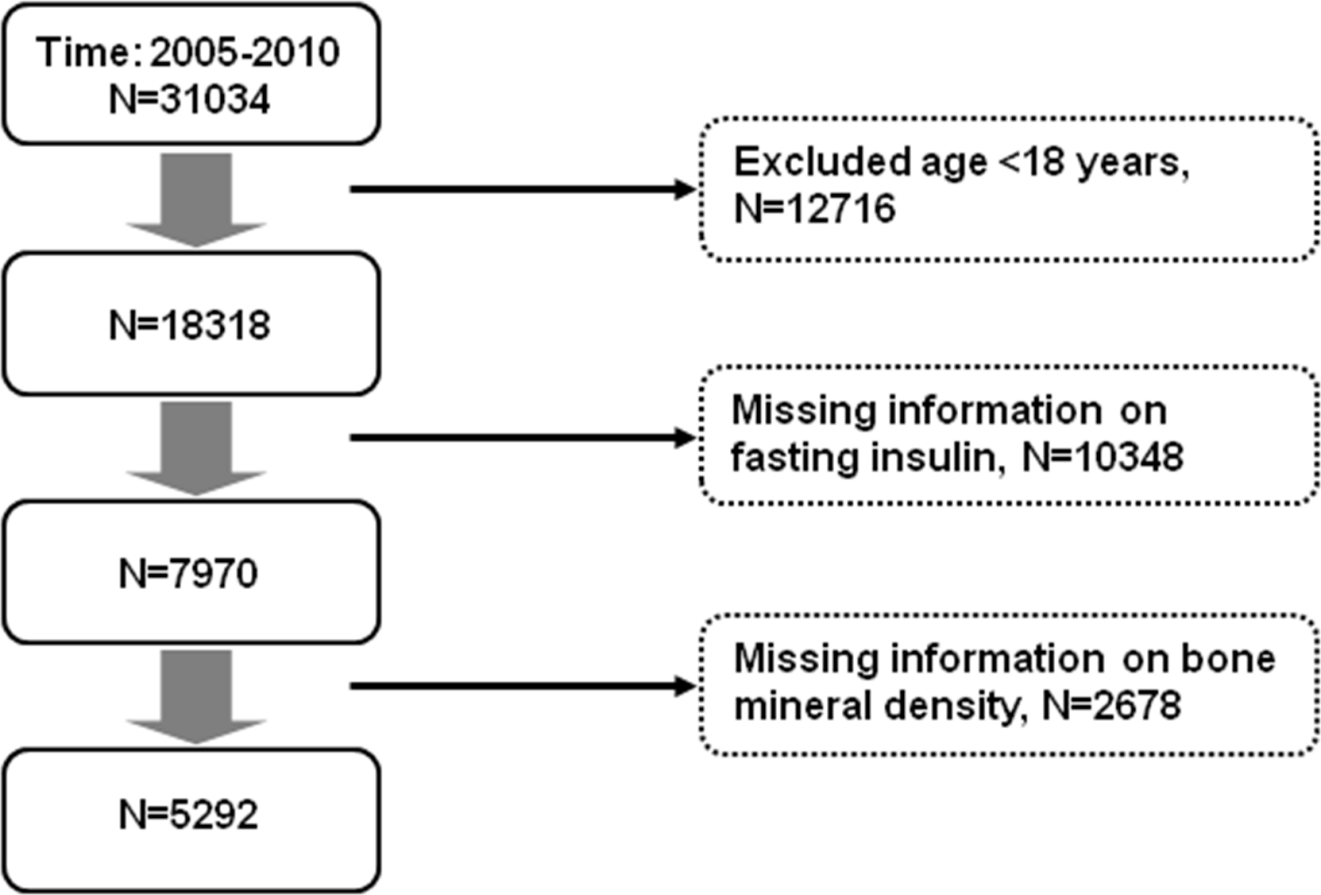
Selection of study participants for analyses.

**Figure 1** shows selection of the study population. Among the 31034 participants in the NHANES from 2005 to 2010, we excluded those aged <18 years or had missing data on fasting insulin or BMD. Finally, we had 5292 participants for analyses. We assessed IR and β-cell secretion of the study population using the Homeostatic Model Assessment (21) for Insulin Resistance (HOMA-IR) and β-cell function (HOMA-β), respectively. HOMA-IR = fasting insulin [μU/l] * fasting glucose [mmol/l]/22.5. HOMA-β = 20 * fasting insulin [μU/l]/(fasting glucose [mmol/l] - 3.5). We divided the study population into four groups according to HOMA-IR (<2 vs. ≥ 2) and HOMA-β (<100 vs. ≥ 100). The cut-off values were decided according to previous studies. Insulin resistance (assessed using HOMA-IR) was associated with low bone mass in men (22) and women (19). The mean HOMA-IR in the high insulin resistance group was 2.0-2.2 (19, 20). In a multi-ethnic study (23) investigating insulin resistance and pancreatic β-cell function, the median HOMA-IR was 1.8-2.2 while the median HOMA-β was 100-120. Hence, we used the cut-off values of HOMA-IR and HOMA-β as 2 and 100, respectively. We used the Chronic Kidney Disease Epidemiology Collaboration equation (24) to determine renal function (estimated glomerular filtration rate, eGFR). An eGFR < 60 ml/min/1.73 m^2^ was considered to be chronic kidney disease. BMD and T score at the lumbar spine, hip joint, and various parts of the femur (femoral neck, greater trochanter, and femoral intertrochanter) were used for analyses. Osteoporosis was defined as a T score ≤ -2.5 (2, 3).

All analyses were conducted using the Statistical Analysis System survey procedures (SAS version 9.4, 2013, Cary, NC, USA). Analysis of Variance (ANOVA) and Chi-square test were used to examine the differences in baseline characteristics, BMD, and T score across the four groups (HOMA-IR <2 and HOMA-β <100, HOMA-IR <2 and HOMA-β ≥ 100, HOMA-IR ≥ 2 and HOMA-β <100, HOMA-IR ≥ 2 and HOMA-β ≥ 100). We used the SAS SURVEYREG procedure to perform the sample-weighted analysis of variance test according to the user’s guide of the analysis program. To examine the associations of HOMA-IR and HOMA-β with osteoporosis, logistic regression analyses were conducted using osteoporosis (T-score ≤ -2.5) as the dependent variable with adjustment for confounding factors (age, sex, race, body mass index, and chronic kidney disease). To examine the joint effects of HOMA-IR and HOMA-β on osteoporosis, logistic regression analyses were conducted using participants with HOMA-IR <2 and HOMA-β <100 as the reference group. Logistic regression was adequately weighted using the SURVEYLOGISTIC procedure. Due to the complex survey design of NHANES, we calculated the weighted data according to analytic guidelines [National Health and Nutrition Examination Survey: Analytic Guidelines, 2011–2014 and 2015–2016 (https://wwwn.cdc.gov/nchs/nhanes/analyticguidelines.aspx)]. A two-sided p value less than 0.05 was considered to be statistically significant in all of the statistical analyses.

## Results

**Table 1** shows the characteristics of the study population according to HOMA-IR and HOMA-β. Participants who had a HOMA-IR ≥ 2 were older and more likely to be male, had a higher body mass index, a higher systolic and diastolic blood pressure, and a higher proportion of having diabetes, compared with those who had a HOMA-IR <2. The former group also had worse metabolic profiles (lower high-density lipoprotein cholesterol, higher triglycerides, fasting plasma glucose, and HbA1c) than the latter.

**Table 1:** Characteristics of the study participants according to HOMA-IR and HOMA-β.

Variables	HOMA-IR <2, HOMA-β <100	HOMA-IR <2, HOMA-β ≥100	HOMA-IR ≥2, HOMA-β <100	HOMA-IR ≥2, HOMA-β ≥100	P-value
N	1856	288	961	2187	
Age, year	43.7 (42.6-44.7)	34.9 (33.4-36.4)	50.2 (48.7-51.6)	42.2 (41.1-43.3)	<0.001
Male, n (%)	993 (53.5)	86 (29.9)	569 (59.2)	1103 (50.4)	<0.001
Race/ethnicity, n (%)					<0.001
Non-Hispanic white	1000 (53.9)	108 (37.5)	420 (43.7)	859 (39.3)	<0.001
Non-Hispanic black	351 (18.9)	74 (25.7)	165 (17.2)	457 (20.9)	<0.001
Mexican American/others	505 (27.2)	106 (36.8)	376 (39.1)	871 (39.8)	<0.001
Body mass index, kg/m2	24.5 (24.3-24.7)	25.9 (25.2-26.6)	28.7 (28.3-29.1)	30.6 (30.2-31.0)	<0.001
Systolic blood pressure, mm Hg	117.8 (117.0-118.7)	113.0 (111.0-115.0)	125.6 (124.3-126.9)	121.2 (120.1-122.3)	<0.001
Diastolic blood pressure, mm Hg	68.5 (67.7-69.4)	65.9 (64.2-67.7)	71.0 (69.8-72.2)	71.0 (70.2-71.9)	<0.001
Smoking, n (%)	846 (45.6)	76 (26.4)	455 (47.3)	861 (39.4)	<0.001
Chronic kidney disease, n (%)^a^	37 (2.0)	2 (0.7)	55 (5.7)	51 (2.3)	<0.001
Hypertension, n (%)	377 (20.3)	37 (12.8)	426 (44.3)	630 (28.8)	<0.001
Diabetes, n (%)	99 (5.3)	4 (1.4)	354 (36.8)	170 (7.8)	<0.001
Total cholesterol, mg/dl	195.4 (192.9-197.8)	190.7 (185.6-195.9)	199.2 (195.7-202.7)	195.6 (193.2-197.9)	<0.001
HDL cholesterol, mg/dl	61.6 (60.8-62.4)	57.2 (55.1-59.3)	51.0 (49.5-52.5)	48.0 (47.4-48.6)	<0.001
Triglycerides, mg/dl	94.1 (89.9-98.4)	96.0 (88.7-103.4)	152.3 (139.1-165.5)	151.4 (144.6-158.1)	<0.001
Fasting plasma glucose, mg/dl	90.9 (89.5-92.2)	79.3 (78.2-80.4)	122.1 (118.4-125.9)	95.5 (94.8-96.2)	<0.001
HbA1c, %	5.3 (5.3-5.4)	5.2 (5.1-5.2)	6.1 (6.0-6.2)	5.4 (5.4-5.5)	<0.001
eGFR, mL/min/1.73 m^2^	97.7 (96.4-98.9)	105.0 (102.4-107.6)	93.4 (91.6-95.3)	100.0 (98.5-101.5)	<0.001
HOMA-IR	1.2 (1.2-1.2)	1.6 (1.5-1.6)	3.8 (3.5-4.0)	5.0 (4.8-5.2)	<0.001
HOMA-β	57.6 (56.4-58.9)	156.6 (128.4-184.7)	72.5 (70.8-74.1)	189.9 (182.6-197.1)	<0.001

Data are presented as mean (95% CI) or n (%). eGFR, estimated glomerular filtration rate. HbA1c, glycated hemoglobin. HDL, high-density lipoprotein. HOMA-IR, homeostasis model assessment-insulin resistance. ^a^eGFR< 60 mL/min/1.73 m^2^.

**Table 2** shows the BMD and T score at various sites (L-spine, hip, and femur) according to HOMA-IR and HOMA-β. Participants with a HOMA-IR ≥ 2 and a HOMA-β ≥ 100 had the highest BMD at L-spine, hip, and femur (all p<0.001) among the four groups. However, they had the lowest BMD at all sites after adjustment for age, sex, race, body mass index, and chronic kidney disease. In contrast, participants with a HOMA-IR <2 and a HOMA-β ≥ 100 had the highest BMD at L-spine, hip, and trochanter and intertrochanter of the femur after adjustment for the confounders. Similar findings were noted regarding the T score (**Table 2**).

**Table 2:** BMD and T score of the study participants according to HOMA-IR and HOMA-β.

	HOMA-IR <2, HOMA-β <100	HOMA-IR <2, HOMA-β ≥100	HOMA-IR ≥2, HOMA-β <100	HOMA-IR ≥2, HOMA-β ≥100	P-value
BMD					
Lumbar spine	1.020 (1.012, 1.027)	1.050 (1.032, 1.068)	1.045 (1.032, 1.057)	1.051 (1.043, 1.059)	<0.001
Total hip	0.951 (0.943, 0.958)	0.986 (0.967, 1.006)	0.989 (0.975, 1.004)	1.019 (1.012, 1.027)	<0.001
Femoral neck	0.823 (0.815, 0.832)	0.869 (0.852, 0.887)	0.846 (0.833, 0.859)	0.884 (0.876, 0.892)	<0.001
Greater trochanter	0.720 (0.713, 0.727)	0.742 (0.726, 0.759)	0.750 (0.737, 0.762)	0.765 (0.757, 0.772)	<0.001
Femoral intertrochanter	1.119 (1.111, 1.127)	1.160 (1.138, 1.183)	1.167 (1.150, 1.183)	1.200 (1.192, 1.209)	<0.001
Adjusted BMD^a^					
Lumbar spine	1.041 (1.028, 1.054)^c^	1.055 (1.036, 1.075)^c^	1.047 (1.032, 1.062)^c^	1.029 (1.014, 1.044)	0.005
Total hip	0.979 (0.969, 0.989)	0.994 (0.976, 1.012)^c^	0.983 (0.970, 0.997)	0.974 (0.962, 0.985)	0.072
Femoral neck	0.854 (0.845, 0.862)	0.859 (0.844, 0.875)	0.860 (0.848, 0.872)	0.848 (0.838, 0.859)	0.211
Greater trochanter	0.735 (0.725, 0.744)^c^	0.746 (0.730, 0.762)^c^	0.738 (0.726, 0.751)	0.726 (0.715, 0.737)	0.008
Femoral intertrochanter	1.155 (1.142, 1.168)	1.175 (1.151, 1.198)^bc^	1.160 (1.143, 1.177)	1.150 (1.137, 1.164)	0.090
T score					
Lumbar spine	-1.503 (-1.565, -1.440)	-1.180 (-1.325, -1.034)	-1.327 (-1.432, -1.221)	-1.248 (-1.315, -1.182)	<0.001
Total hip	-0.306 (-0.353, -0.259)	0.129 (-0.000, 0.258)	-0.083 (-0.182, 0.016)	0.202 (0.144, 0.261)	<0.001
Femoral neck	-0.564 (-0.623, -0.505)	-0.090 (-0.214, 0.035)	-0.432 (-0.531, -0.334)	-0.098 (-0.162, -0.034)	<0.001
Greater trochanter	-0.246 (-0.302, -0.191)	0.103 (-0.030, 0.236)	-0.034 (-0.138, 0.070)	0.162 (0.093, 0.230)	<0.001
Femoral intertrochanter	-0.207 (-0.252, -0.163)	0.236 (0.106, 0.366)	0.026 (-0.073, 0.124)	0.304 (0.250, 0.358)	<0.001
Adjusted T score^a^					
Lumbar spine	-1.323 (-1.430, -1.216)^c^	-1.205 (-1.369, -1.041)^c^	-1.277 (-1.401, -1.153)^c^	-1.423 (-1.548, -1.298)	0.005
Total hip	-0.101 (-0.176, -0.025)	0.020 (-0.115, 0.155)^bc^	-0.060 (-0.162, 0.043)	-0.129 (-0.216, -0.042)	0.071
Femoral neck	-0.333 (-0.399, -0.267)	-0.280 (-0.399, -0.161)	-0.278 (-0.369, -0.186)	-0.368 (-0.452, -0.284)	0.191
Greater trochanter	-0.122 (-0.210, -0.033)	-0.011 (-0.161, 0.138)^c^	-0.078 (-0.192, 0.035)	-0.187 (-0.288, -0.087)	0.014
Femoral intertrochanter	0.017 (-0.064, 0.098)	0.154 (0.001, 0.306)^bc^	0.057 (-0.053, 0.168)	-0.002 (-0.090, 0.087)	0.084

Data are presented as mean (95% CI). BMD, bone mineral density. HOMA-IR, homeostasis model assessment-insulin resistance. ^a^Adjusted for age, sex, race, body mass index, and chronic kidney disease. ^b^p<0.05 vs. HOMA-IR <2, HOMA-β <100. ^c^p<0.05 vs. HOMA-IR ≥2, HOMA-β ≥100.

The association between HOMA-IR and osteoporosis is shown in **Table 3**. Overall, there was no significant association between HOMA-IR and osteoporosis. The findings were similar in participants with a HOMA-β <100. Nevertheless, we observed a positive association between HOMA-IR and osteoporosis in participants who had a HOMA-β ≥ 100, especially at the femoral neck (OR 8.773, 95% CI 2.160-35.637, p=0.002, p interaction 0.010, (**Table 3**).

**Table 3:** Association of HOMA-IR with osteoporosis^a^.

	Overall		HOMA-β <100		HOMA-β ≥100		
	OR (95% CI)^b^	P	OR (95% CI)^b^	P	OR (95% CI)^b^	P	P for interaction
Lumbar spine	1.109 (0.903-1.362)	0.325	1.047 (0.781-1.403)	0.761	1.248 (0.785-1.984)	0.349	0.176
Total hip	0.788 (0.396-1.568)	0.498	0.553 (0.227-1.347)	0.192	2.601 (0.780-8.670)	0.120	0.188
Femoral neck	1.270 (0.834-1.934)	0.265	1.072 (0.669-1.717)	0.774	8.773 (2.160-35.637)	0.002	0.010
Greater trochanter	1.230 (0.584-2.589)	0.586	0.935 (0.396-2.203)	0.877	3.016 (0.439-20.726)	0.262	0.238
Femoral intertrochanter	0.654 (0.298-1.437)	0.291	0.492 (0.167-1.454)	0.200	2.851 (0.212-38.264)	0.429	0.089

HOMA-IR, homeostasis model assessment-insulin resistance. ^a^T score ≤ -2.5. ^b^Adjusted for age, sex, race, body mass index, and chronic kidney disease.

**Table 4** shows the association between HOMA-β and osteoporosis. There was no significant association between HOMA-β and osteoporosis in the overall population. In contrast to the non-significant association in participants who had a HOMA-IR ≥ 2, we observed a negative association between HOMA-β and osteoporosis in participants with a HOMA-IR <2 (especially at the femoral neck, OR 0.183, 95% CI 0.038-0.882, p=0.034, p interaction 0.010, **Table 4**).

**Table 4:** Association of HOMA-β with osteoporosis^a^.

	Overall		HOMA-IR <2		HOMA-IR ≥2		
	OR (95% CI)^b^	P	OR (95% CI)^b^	P	OR (95% CI)^b^	P	P for interaction
Lumbar spine	1.107 (0.887-1.382)	0.369	0.876 (0.495-1.552)	0.650	1.128 (0.873-1.457)	0.358	0.176
Total hip	0.959 (0.474-1.939)	0.906	0.554 (0.130-2.350)	0.423	1.208 (0.603-2.420)	0.594	0.188
Femoral neck	1.128 (0.605-2.105)	0.704	0.183 (0.038-0.882)	0.034	1.118 (0.505-2.474)	0.783	0.010
Greater trochanter	1.123 (0.608-2.076)	0.711	0.473 (0.130-1.728)	0.258	1.197 (0.555-2.581)	0.647	0.238
Femoral intertrochanter	0.865 (0.447-1.673)	0.667	0.250 (0.033-1.888)	0.179	1.386 (0.650-2.957)	0.398	0.089

HOMA-IR, homeostasis model assessment-insulin resistance. ^a^T score ≤ -2.5. ^b^Adjusted for age, sex, race, body mass index, and chronic kidney disease.

**Table 5** shows the joint effect of HOMA-IR and HOMA-β on osteoporosis. Compared with participants who had HOMA-IR <2 and HOMA-β <100, those with HOMA-IR <2 and HOMA-β ≥ 100 had a lower risk of osteoporosis (OR 0.126, 95% CI 0.020-0.805, p=0.032 at the femoral neck). This was not the case in participants with HOMA-IR ≥ 2. With regard to osteoporosis at the femoral neck, HOMA-IR ≥ 2 was associated with a higher risk compared with HOMA-IR <2 and HOMA-β ≥ 100. The risk was even higher in participants with HOMA-IR ≥ 2 and HOMA-β ≥ 100 (**Table 5**).

**Table 5:** Joint effect of HOMA-IR and HOMA-β on osteoporosis^a^.

	HOMA-IR <2, HOMA-β <100	HOMA-IR <2, HOMA-β ≥100	HOMA-IR ≥2, HOMA-β <100	HOMA-IR ≥2, HOMA-β ≥100
Lumbar spine	Ref	0.826 (0.457-1.493)	0.986 (0.755-1.287)	1.145 (0.897-1.460)
Total hip	Ref	0.433 (0.091-2.053)	0.599 (0.259-1.389)	0.766 (0.344-1.706)
Femoral neck	Ref	0.126 (0.020-0.805)	1.070 (0.656-1.744)^b^	1.256 (0.625-2.526)^b^
Greater trochanter	Ref	0.427 (0.103-1.778)	1.036 (0.438-2.448)	1.278 (0.554-2.945)
Femoral intertrochanter	Ref	0.225 (0.028-1.796)	0.493 (0.175-1.392)	0.827 (0.381-1.796)

Data are presented as OR (95% CI), adjusted for age, sex, race, body mass index, smoking, hypertension, and chronic kidney disease. HOMA-IR, homeostasis model assessment-insulin resistance. ^a^T score ≤ -2.5. ^b^p<0.05 vs. HOMA-IR <2, HOMA-β ≥100.

## Discussion

In this cross-sectional study using data from the NHANES, we demonstrated that HOMA-β ≥ 100 with HOMA-IR <2 was associated with a higher BMD and T score, as well as a lower risk of osteoporosis (vs. HOMA-β <100 with HOMA-IR <2). In contrast, HOMA-β ≥ 100 with HOMA-IR ≥ 2 was associated with lower BMD and T scores (vs. HOMA-β ≥ 100 with HOMA-IR <2). Our findings suggest that the association between insulinemia and BMD/T score might be different depending on an individual’s IR status.

Previous studies revealed that insulin plays an important role in the anabolic effect on bone mass and trabecular bone microarchitecture (15, 16, 25, 26). Increased proliferation and collagen synthesis in response to insulin treatment were noted in *in vitro* studies using cultured osteoblasts (27–29). Moreover, insulin may exert synergistic effects with insulin-like growth factor 1 and parathyroid hormone (30, 31), both of which have anabolic effects on bone cells. These findings are in line with previous studies using insulin-deficient animal models in which reduced bone formation was noted (32, 33). Furthermore, insulin treatment might reverse the deficiency (34, 35). The aforementioned results are supported by the findings of decreased bone mass in patients with type 1 diabetes (36–38).

Nevertheless, there are conflicting findings in type 2 diabetes. People with type 2 diabetes have a higher BMD, but a higher risk of fracture, than those without the disease (39–41). This phenomenon suggests that IR may influence the effects of insulinemia on bone mass. Shin D, et al. (22) reported different associations between insulinemia and BMD at various levels of HOMA-IR in men. Consistent with our findings, fasting insulin level was positively associated with BMD at low HOMA-IR. However, a negative association was observed at a higher IR state. Similarly, fasting insulin was positively associated with BMD in adolescents in a cross-sectional study (42). Nevertheless, an inverse association was noted after adjustment for fat mass. Hence, IR may affect the association between insulinemia and bone mass, and investigations on different populations may yield inconsistent results (15–20). We examined the joint effect of HOMA-IR and HOMA-β on osteoporosis (**Table 5**). Our findings suggest that HOMA-β ≥ 100 with HOMA-IR <2 was associated with a lower risk of osteoporosis. In contrast, the risk increased with HOMA-β ≥ 100 and HOMA-IR ≥ 2.

It is interesting to note that the associations among HOMA-β, HOMA-IR, and BMD/osteoporosis were more apparent at the femoral neck (**Table 3**–**5**). IR has been negatively associated with cortical bone volume and bone strength at the femoral neck in postmenopausal women (18). Similar findings were noted in a general population (43), although the negative association between IR and BMD at the femoral neck became non-significant after adjustment for body mass index. The associations among HOMA-IR, HOMA-β, and BMD/osteoporosis at different anatomic sites merit further investigation (44).

Our study had several limitations. First, we investigated the associations among HOMA-IR, HOMA-β, and BMD/osteoporosis using cross-sectional data. Thus, the causal relationships could not be confirmed. Second, some relevant factors (such as diet and exercise) were not analyzed. This might have confounded our results. Third, we did not have data on fracture events. We could not confirm that the associations among HOMA-IR, HOMA-β, and BMD/osteoporosis were linked to risk of fracture. Despite these limitations, we demonstrated a joint effect of HOMA-IR and HOMA-β on osteoporosis which may help explain the inconsistent findings in populations with high variations in IR and pancreatic β-cell function (15–20, 45).

In conclusion, the association between insulinemia (HOMA-β) and BMD/osteoporosis changed as IR (HOMA-IR) increased. HOMA-β was negatively associated with osteoporosis at a low level of HOMA-IR (<2). The association was not significant when HOMA-IR ≥ 2. The mechanisms by which IR affects the association between insulinemia and osteoporosis merit further investigation.

## Data availability statement

The datasets presented in this study can be found in online repositories. The names of the repository/repositories and accession number(s) can be found below: https://wwwn.cdc.gov/nchs/nhanes/Search/DataPage.aspx?Component=Dietary&CycleBeginYear=2005.

## Ethics statement

The studies involving human participants were reviewed and approved by Institutional Review Board of Taichung Veterans General Hospital, Taichung, Taiwan. The patients/participants provided their written informed consent to participate in this study.

## Author contributions

C-LL and J-SW contributed to conception and design of the study. Y-HF and W-JL organized the database. W-JL and C-LL performed the statistical analysis. Y-HF and J-SW wrote the first draft of the manuscript. W-JL and C-LL reviewed and edited the manuscript. All authors contributed to the article and approved the submitted version.

## Funding

This research was funded by Taichung Veterans General Hospital [grant number TCVGH-1093504C, TCVGH-1097316C, TCVGH-1097327D, TCVGH-1103502C, and TCVGH-1113502C]. The sponsors had no role in the design, execution, interpretation, or writing of the study.

## Acknowledgments

We would like to thank the participants in the NHANES, and the members of the National Center for Health Statistics for collecting the data and making it publicly available.

## Conflict of interest

The authors declare that the research was conducted in the absence of any commercial or financial relationships that could be construed as a potential conflict of interest.

## Publisher’s note

All claims expressed in this article are solely those of the authors and do not necessarily represent those of their affiliated organizations, or those of the publisher, the editors and the reviewers. Any product that may be evaluated in this article, or claim that may be made by its manufacturer, is not guaranteed or endorsed by the publisher.
